# Anatomy and relationships of forelimb lymph nodes in Sprague-Dawley rats: A detailed dissecting approach

**DOI:** 10.3389/fvets.2022.912278

**Published:** 2022-08-25

**Authors:** Linhai Chen, Jing Yang, Sang Ah Kim, Ma. Nessa Gelvosa, Peng Wei, Jae Yong Jeon, Hwayeong Cheon

**Affiliations:** ^1^Department of Plastic and Reconstructive Surgery, Ningbo First Hospital, Ningbo, China; ^2^Department of Anesthesiology, The First Affiliated Hospital of Zhejiang University Medical College, Hangzhou, China; ^3^Department of Rehabilitation Medicine, Asan Medical Center, University of Ulsan College of Medicine, Seoul, South Korea; ^4^Biomedical Engineering Research Center, Asan Medical Center, Asan Institute for Life Sciences, Seoul, South Korea

**Keywords:** animal model for lymphedema research, lymphatic anatomy, lymph node flap in the forelimb, Sprague-Dawley rat, surgical procedure

## Abstract

**Background:**

Constructing a reliable animal model for preclinical treatment of secondary lymphedema is challenging because the anatomical characteristics near the lymph nodes are understudied. Therefore, this study examined the detailed anatomical relationship between the axillary lymph node flaps (ALNFs) and brachial lymph node flaps (BLNFs) in the forelimb of Sprague-Dawley (SD) rats.

**Materials and methods:**

Ten male rats, weighing 250–300 g, were used. The ALNFs and BLNFs on either side of the rat forelimbs were dissected. The two lymph node flaps (LNFs) were immediately harvested to analyze their physical characteristics (via imaging process software) and microscopic structure (via histology examinations).

**Results:**

A total of 20 ALNFs and BLNFs from 10 rats were harvested and analyzed. ALNF dissection was simpler and lasted a shorter time than BLNF dissection (*p* < 0.0001). The left LNFs were more difficult to dissect than the right LNFs (*p* < 0.0001). In physical characteristics of LNFs, the area (*p* < 0.001) of LNFs and the number of lymph nodes (*p* < 0.0001) associated with ALNFs were greater than those associated with BLNFs, but the pedicle lengths of ALNFs were shorter than that of BLNFs (*p* < 0.0001). No significant difference in the diameter of the venous and arterial pedicles was noted between the two LNFs (*p* > 0.05).

**Conclusion:**

This study reported detailed physical characteristics of ALNFs and BLNFs in SD rat forelimbs, assessing the respective area of LNFs, number of lymph nodes, and lengths and diameters of vascular pedicles. Moreover, this study suggested an efficient method to perform a study of LNFs by describing the operation process and repeatedly measuring the operation time.

## Introduction

Lymphedema is a set of chronic diseases, affecting ~250 million people worldwide. It is caused by the accumulation of high-protein interstitial fluid that results from local tissue proliferation and inflammation, induced by diseases of the lymphatic system (1). Although there is no effective cure for lymphedema thus far, some breakthroughs have been made to improve treatment outcomes. In particular, the effect of certain physiological surgical treatments (such as autologous vascularized lymph node transfer and lymphovenous anastomosis, on moderate to severe lymphedema) has been significant, based on staging outlines from the International Society of Lymphology (2). However, the mechanism of these surgeries and their effect on the pathophysiology of lymphedema remain unclear and require further exploration (3).

Given that lymphedema is a complex disease involving multiple tissue components, developing preclinical *in*–*vitro* programs to understand its pathophysiology is challenging (4). The development of an appropriate animal model is thus meaningful. Despite many animal models having been established for lymphedema experiments, rats have been the predominant animal used in these models, due to the availability, low cost, and easy manipulation associated with these animals (5). Both the lymphatic territories of rats (6) and specific anatomical steps to accurately dissect lymph nodes need to be understood to achieve optimal experimental objectives.

This study aims to explain, in detail, how to obtain two lymph node flaps (LNFs)—the axillary lymph node flaps (ALNFs) and brachial lymph node flaps (BLNFs)—in a rodent model, and describe the anatomical relationship between these LNFs in the Sprague–Dawley (SD) rat forelimb. Furthermore, this study collected and analyzed two–dimensional information and pathological data associated with these two LNFs, since their physical characteristics have rarely been studied.

## Materials and methods

This study was reviewed and approved by the Institutional Animal Care and Use Committee (IACUC) of the Asan Institute for Life Sciences, Asan Medical Center in Republic of Korea. The IACUC abides by the Institute of Laboratory Animal Resources (ILAR) guide. Ten male SD rats, weighing 250–300 g, were used in the study. Because there was no difference in the anatomical structure of lymphatics in the forelimb and hindlimbs between the sexes (7, 8), we used male rats that were higher resistant to surgical trauma in long–term operations. Before the operation, all rats were further anesthetized with a mixture of Zoletil (Tiletamine–Zolazepam, Virbac, France; 10 ml/kg) and Rumpun (xylazine hydrochloride, Bayer Korea, Republic of Korea; mixing volume ratio, 5:1), after being induced using 4% isoflurane gas. Then, the backs of the rats were shaved using an electric razor and depilatory cream. During the experiment, the animals were kept in separate cages, at a constant temperature of 20°C. At the end of the experiment, the anesthetized rats were euthanized by inhaling carbon dioxide gas for 7 min, in accordance with the American Veterinary Medical Association guidelines for the euthanasia of animals.

### Surgical approach to dissect ALNF and BLNF

**Figure 1:**
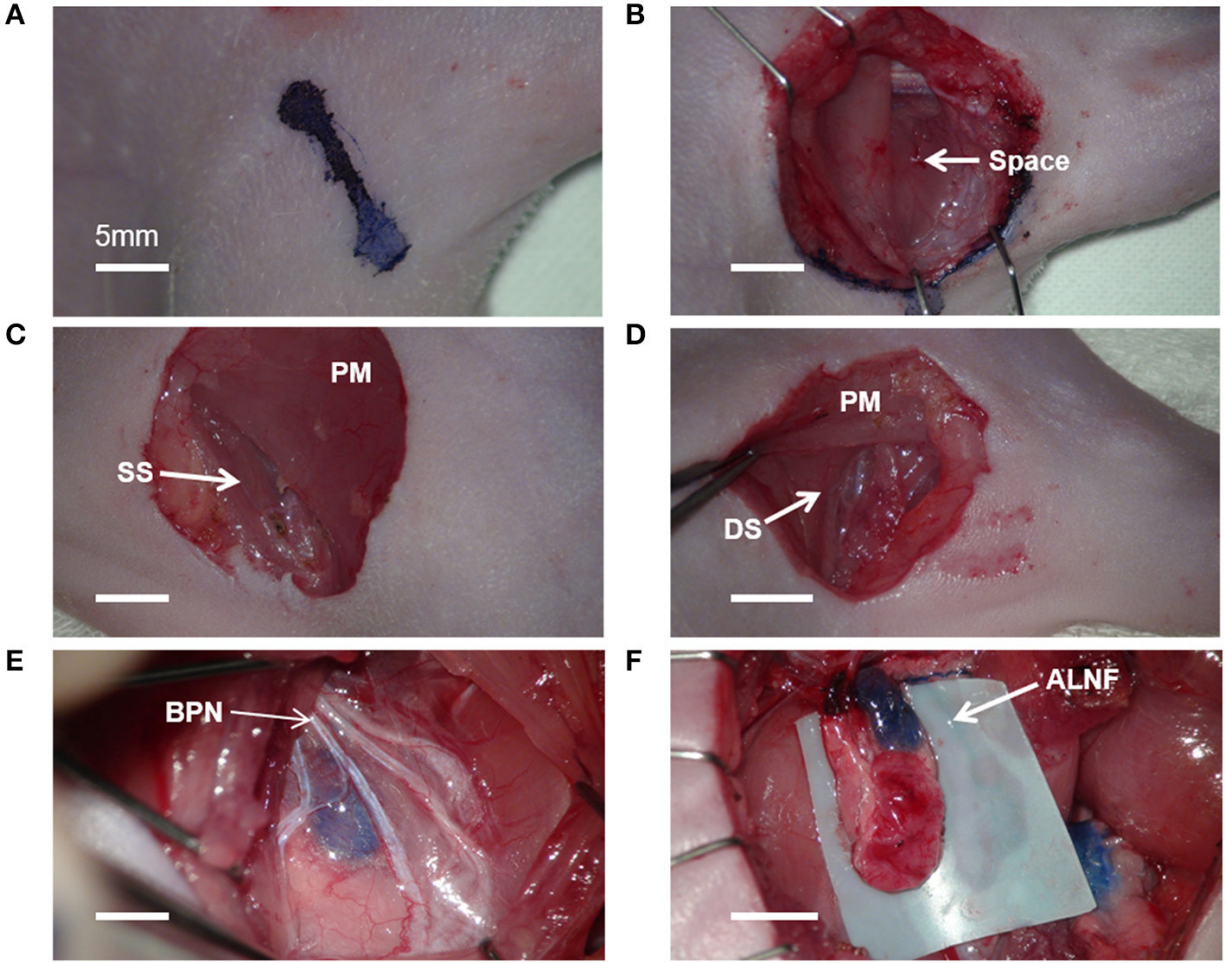
Microsurgical procedure for axillary lymph node flaps (ALNFs). **(A)** Axillary incision, 1–1.5 cm. **(B)** The space between the proximal forelimb and lateral chest. **(C)** Dissecting approach to the superficial surface (SS) of the pectoralis major (PM). **(D)** Dissecting approach to the deep surface (DS) of the PM. **(E)** The branches of the brachial plexus nerve (BPN), exposed above the ALNF. **(F)** Complete elevation of the ALNF.

After anesthesia, the rats were placed in a supine position on the operating table. Methylene blue (15 mg/mL aqueous solution, Sigma, MO, USA) of 0.05 ml was injected subcutaneously into the palms of all rats to visualize the lymphatics. The temperature in the operation room was maintained at 20°C during the surgical procedure. Because the lymphatic structure of the rat is very small, we used a microsurgery system (F170, Carl Zeiss, Germany), the 5–mm curved micro–dissecting spring scissors (Strub Medical, Germany), the microsurgical blades (Medline Industrie, IL, USA), and an electrosurgical cautery (Symmetry Surgical TN, USA). An axillary incision of 1.0–1.5 cm was made on one side of the rat *via* microsurgery (Figure 1A). Following blunt dissection through the exposed subcutaneous fat, the space between the proximal forelimb and the lateral chest was identified. The lateral border of the pectoralis major has been identified anteriorly, and the dorsal side of the latissimus dorsi was positioned posteriorly through the space (Figure 1B). Identification of these anatomical features was the most critical step during dissection.

**Figure 2:**
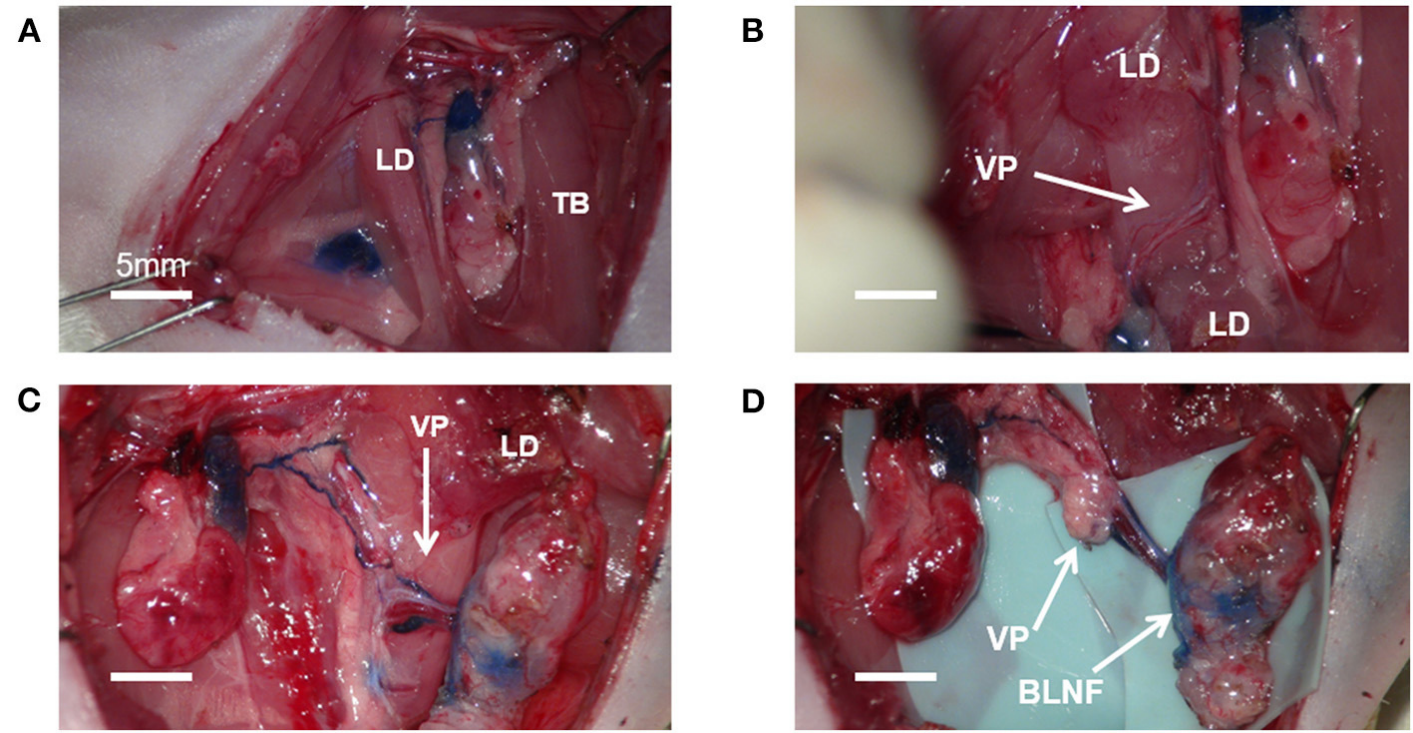
Microsurgical procedure for brachial lymph node flaps (BLNFs). **(A)** BLNF is located between the triceps brachii (TB) and latissimus dorsi (LD). **(B)** The vascular pedicle (VP) was exposed by cutting part of the latissimus dorsi. **(C)** A complete release of the vascular pedicle in BLNF. **(D)** Complete elevation of the BLNF.

Given that ALNFs are concealed in the deep surface of the pectoralis major, two dissecting approaches can be used. One approach involves incising the muscle fibers in parallel on the superficial surface of the muscle (about 5 mm medial to the lateral edge of the muscle; Figure 1C); the other approach involves cutting the muscle fibers in parallel after exposing the deep surface of the muscle by a retractor (Figure 1D). Of the two, the first approach was deemed simpler and more appropriate. For the first approach, the branches of the brachial plexus were gently stripped above the LNFs, to reduce iatrogenic damage (Figure 1E). Once the blue-stained lymph nodes were observed, the medial edge of the LNF was first peeled off; then, the LNF was raised to separate the deep surface, followed by the lateral edge. Thereafter, the two small vascular pedicles at the distal end of the LNF were cauterized. Finally, after the LNF was completely elevated, the pedicle was carefully skeletonized, and the pedicle is composed of the lateral artery and vein (Figure 1F). Similarly, the BLNFs were located in the inferior, deep part of the space between the triceps brachii and latissimus dorsi (Figure 2A). In contrast to that of ALNFs, the pedicles of the BLNFs were dissected first, since they penetrated the latissimus dorsi. Cutting the latissimus dorsi allowed to increase the operation space and also protected the vascular pedicle from being damaged during deep operation (Figure 2B). In addition, BLNFs were close to the dorsal skin, where many small branches to the dermis could be seen. These branches can be easily cauterized under a large operating field of view. Hence, BLNFs were lifted as quickly and safely as possible after the pedicle was completely released (Figure 2C). The pedicle is made up of the thoracodorsal artery and veins. (Figure 2D). During the dissection, the time required to harvest ALNFs and BLNFs was recorded. All rats were operated on by the same surgeon.

### Two–dimensional measurement of LNFs

The harvested LNF tissue was immediately photographed under 4 × enlargements, using a magnifying loupe. Based on the size of the reference objects (including the length and diameter of the pedicle, and the area of the LNFs), two–dimensional data of the flaps were measured and analyzed, using ImageJ software (1.48v; NIH, Bethesda, MD).

### Identification of the number of lymph nodes in LNFs using histological examination

After the measurement of the characteristics in fresh LNFs, the samples were fixed with 4% paraformaldehyde and embedded in paraffin. Paraffin sections were cut into 4–μm slices and stained with hematoxylin and eosin (H&E staining). All images of histological slides were acquired using a light microscope (Model BX40; Olympus, Tokyo, Japan). The number of lymph nodes was counted for each LNF from a sufficient number of slides using the images at 40 × magnification.

### Statistical analysis

The continuous data were presented as mean ± standard deviation. Analyses were performed using GraphPad Prism 9 (GraphPad Software, Inc., San Diego, CA) and Microsoft Excel (version 16.44; Microsoft Corporation). Student's t-tests and one-way ANOVA were used to detect significant differences between LNFs characteristics. *p* < 0.05 was considered significant. All sample data was tested by the Shapiro-Wilk normality test using R-4.2.1 for Windows and they satisfied normality (normality *p*>0.05) except the pedicles in both sides of ALNF and BLNF.

## Results

### Operation time

**Figure 3:**
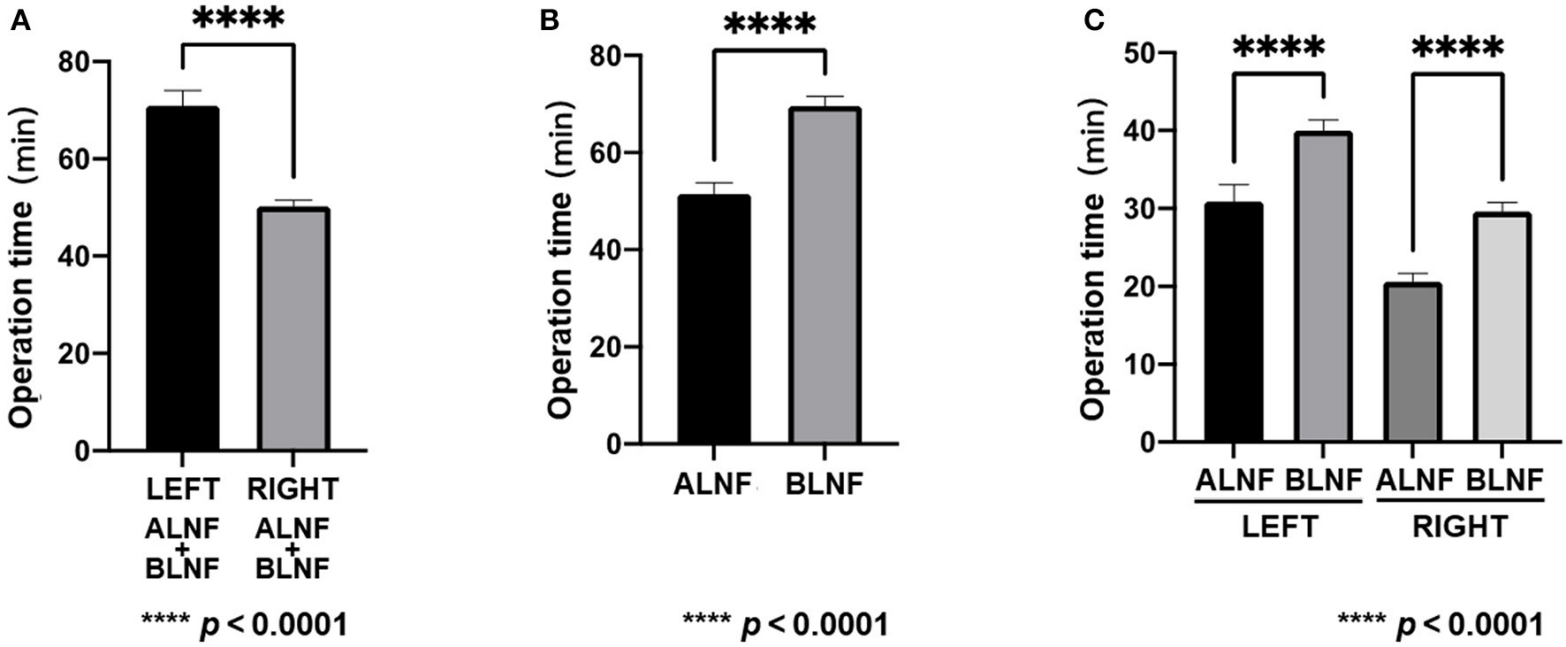
Operation time. **(A)** Comparison of the total time for dissection of the right and left flaps. **(B)** Comparison of total dissection time of ALNF with that of BLNF. **(C)** Comparison of ALNF and BLNF on the same side. **** indicate the p-value between each group (*p* < 0.0001).

A total of 20 LNFs were harvested from each of the ALNFs and BLNFs in the rat's forelimbs. No rats died during the procedure. The operation time was defined as the time from skin incision to completion of LNF dissection. The operation was performed only by dissection of the lymph nodes, lymph vessels, and pedicles related to the LNFs while minimizing damage to the surrounding organs. The total time for dissection of the left forelimb LNF was 70.9 ± 3.2 min, while the total time for dissection of the right forelimb LNF was 50.2 ± 1.3 min (Figure 3A). The variance of the data was statistically significant in both groups (*p* < 0.0001). Furthermore, the total time for dissecting ALNFs (51.5 ± 2.3 min) was significantly shorter than that of BLNFs (69.6 ± 2.0 min; *p* < 0.0001; Figure 3B). In addition, regarding ipsilateral comparisons, the time for dissecting ALNFs was shorter than that of BLNFs (*p* < 0.0001; Figure 3C): for the right side, dissection of ALNFs and BLNFs lasted 20.6 ± 1.1 min and 29.6 ± 1.2 min long, respectively; for the left side, dissection of ALNFs and BLNFs lasted 30.9 ± 2.2 min and 40.2 ± 1.3 min long, respectively.

### Area of LNFs in rats

**Figure 4:**
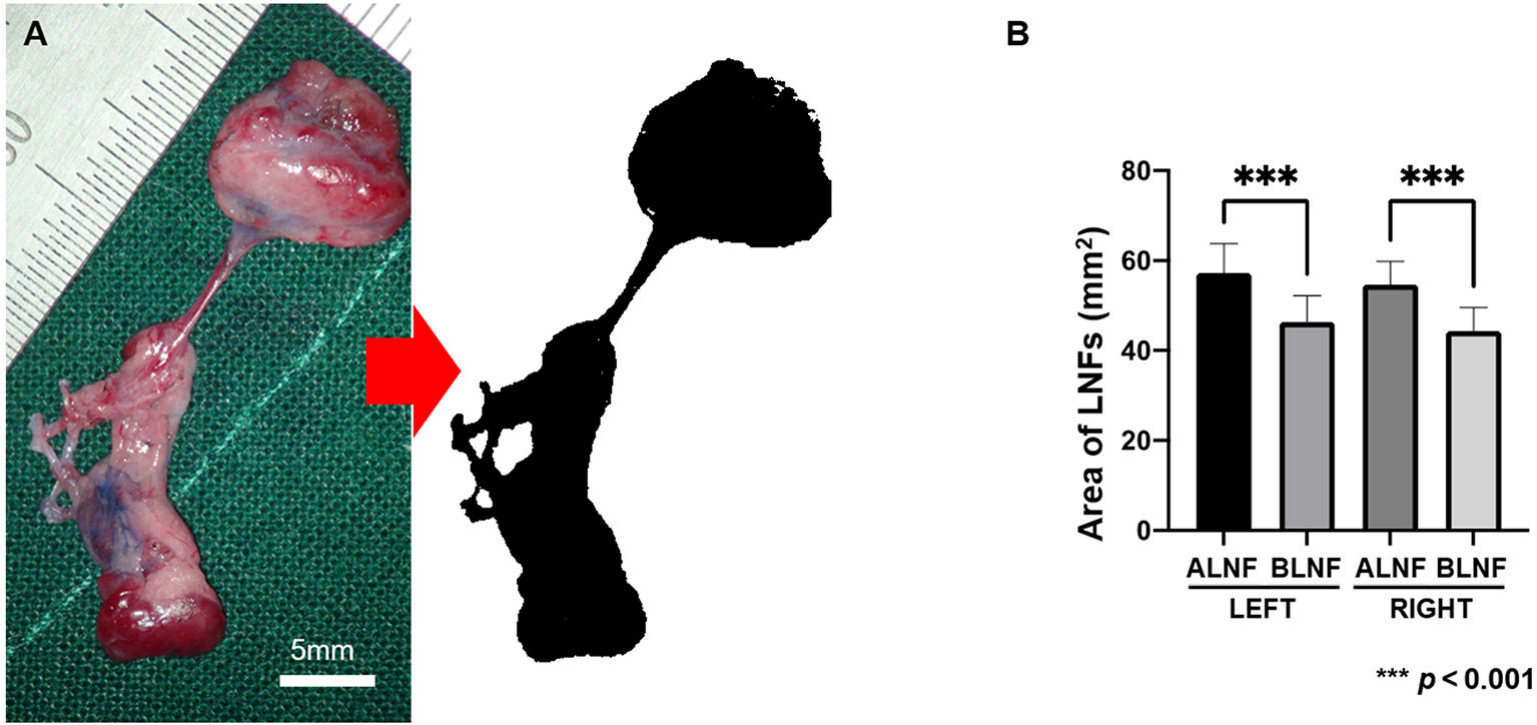
**(A)** The harvested LNF tissue photographed under 4 × enlargements and the image processed to measure the area (black area). The scale bar is 5 mm. **(B)** Area of LNFs on each side. In this bar chart, the difference in area between the ipsilateral ALNF and BLNF is compared; the area of the same type of flap on different sides is compared. *** indicate the p-value between each group (*p* < 0.001).

The left areas of the ALNFs and BLNFs were 50.26 ± 6.51 mm^2^ and 46.38 ± 5.86 mm^2^, respectively, while that of the right areas of ALNFs and BLNFs were 54.68 ± 5.17 mm^2^ and 44.33 ± 5.24 mm^2^, respectively. ALNFs area was significantly larger than the BLNFs area (*p* < 0.001; Figure 4). However, the difference between the left and right sides of the same LNFs was not statistically significant (both *p* > 0.05).

### Vascular pedicle length and diameter of the LNFs

**Figure 5:**
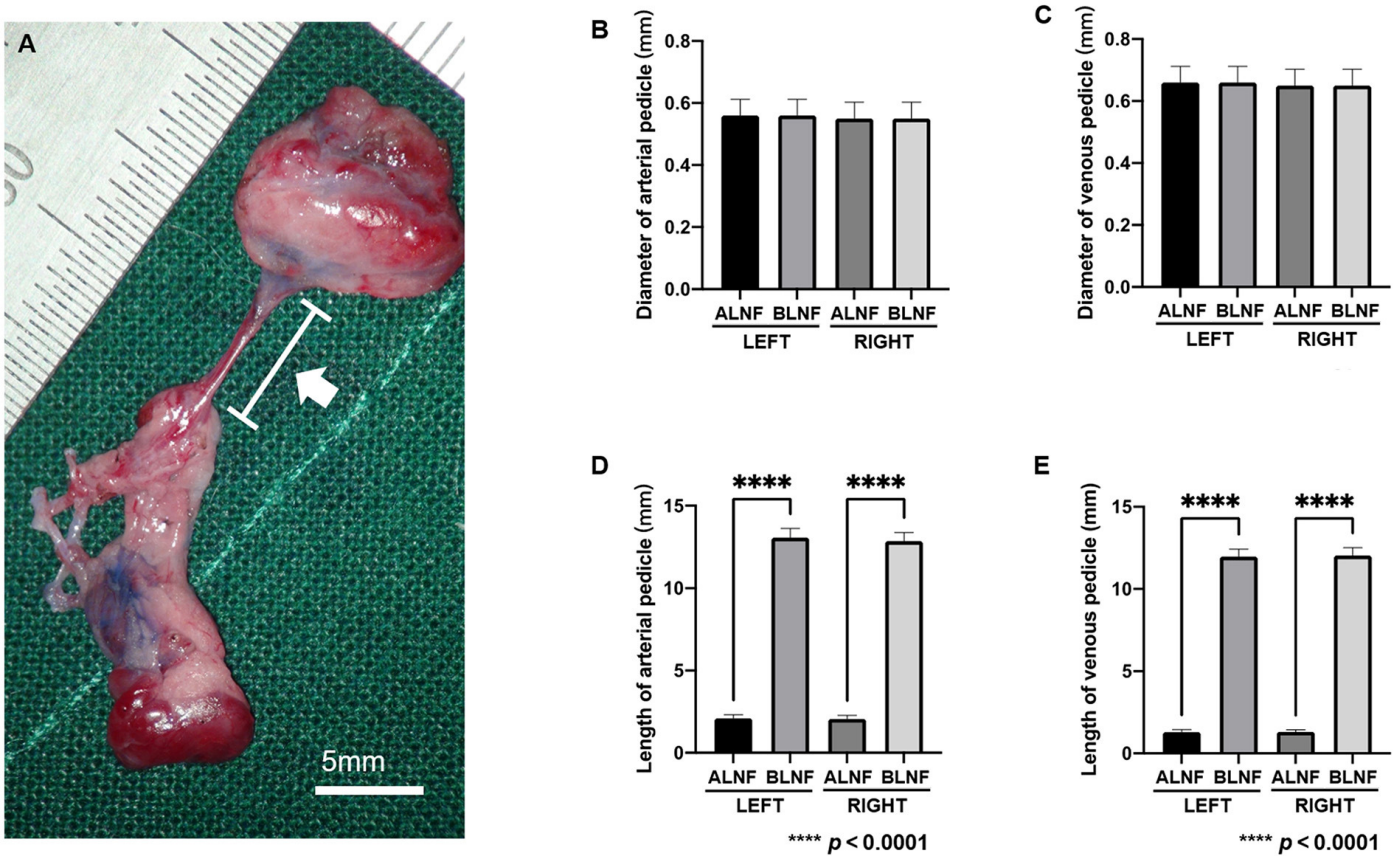
**(A)** Photographic image for the vascular pedicle in the harvested LNFs (white arrow). The scale bar is 5 mm. **(B)** The measurement data of the length and diameter of the vascular pedicle of the LNFs. Comparison of arterial pedicle diameter among groups. **(C)** Comparison of venous pedicle diameter among groups. **(D)** The difference in the length of the arterial pedicle between the ipsilateral ALNF and BLNF is compared; the length of the arterial pedicle of the same type of flap on different sides is compared. **(E)** The difference in the length of the venous pedicle between the ipsilateral ALNF and BLNF is compared; the length of the venous pedicle of the same type of LNF on different sides is compared. **** indicate the p-value between each group (*p* < 0.0001).

In the ALNFs area, the lengths of the left and right arterial pedicles were 2.09 ± 0.22 mm and 2.06 ± 0.22 mm, respectively, while the diameters of the left and right pedicles were 0.56 ± 0.05 mm and 0.55 ± 0.05 mm, respectively. For BLNFs, the lengths of the left and right arterial pedicles were 13.07 ± 0.56 mm and 12.85 ± 0.53 mm, respectively, and the diameters of the left and right pedicles were 0.55 ± 0.05 mm and 0.55 ± 0.05 mm, respectively (*p* < 0.001; Figures 5A–C). In the comparison of the venous pedicle, pedicle lengths of the left and right ALNFs were 1.29 ± 0.16 mm and 1.30 ± 1.33 mm, respectively, and pedicle diameters of the left and right ALNFs were 0.66 ± 0.05 mm and 0.65 ± 0.05 mm, respectively. Meanwhile, pedicle lengths of the left and right BLNFs were 11.97 ± 0.45 mm and 13.03 ± 0.48 mm, respectively, and pedicle diameters of the left and right BLNFs were 0.65 ± 0.05 mm and 0.65 ± 0.05 mm, respectively. No significant difference was detected in the diameter of venous vs. arterial pedicles between the two LNFs in ANOVA test (*p* > 0.05; Figures 5D,E). Pedicle lengths of BLNFs were significantly longer than that of ALNFs (*p* < 0.0001). No statistically significant difference in pedicle length was found when examining different sides in ANOVA test (*p* >0.05; Figures 5D,E).

### The number of lymph nodes in ALNFs and BLNFs

**Figure 6:**
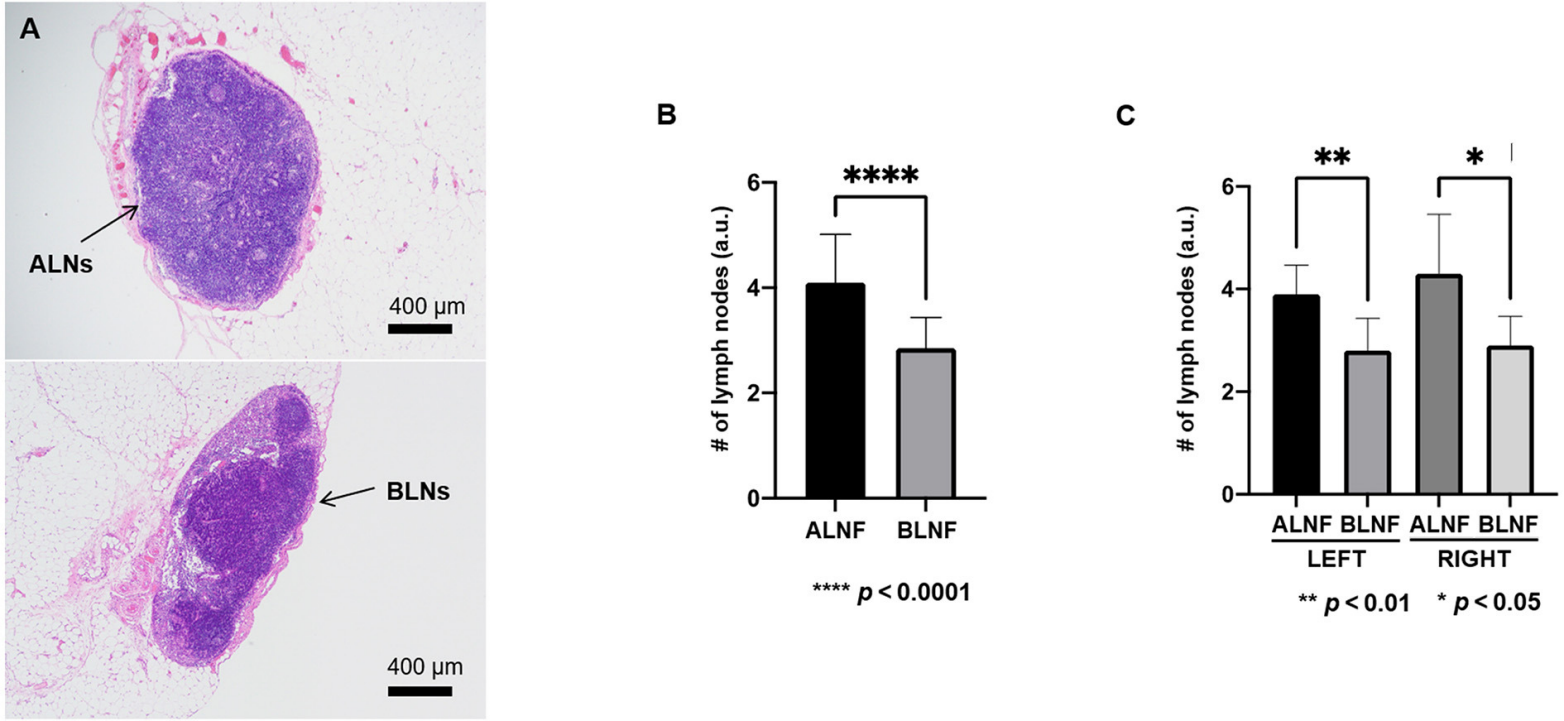
**(A)** In the image magnified 40 × after H&E staining, the typical pathological image of axillary lymph nodes (ALNs) and brachial lymph nodes (BLNs) in the vascularized lymph node flap. **(B)** The number of lymph nodes in ALNF and BLNF. **(C)** The difference in the number of lymph nodes between the ipsilateral ALNF and BLNF is compared; the number of lymph nodes of the same type of LNFs on different sides is compared. ****, **, and * indicate the p-value between each groups (**** *p* < 0.0001, ** *p* < 0.01, * *p* < 0.05).

We analyzed several sections of the LNFs in H&E staining to determine the number of lymph nodes constituting the LNFs (Figure 6A). As the results, 4.10 ± 0.91 lymph nodes and 2.85 ± 0.59 lymph nodes were found on each of the ALNFs and BLNFs, respectively (*p* < 0.0001; Figure 6B). Differences in the number of lymph nodes were found on the same sides (left vs. right, *p* = 0.005 vs. *p* = 0.019; Figure 6C), but no differences in the number of lymph nodes were found between different sides (*p* > 0.05).

## Discussion

SD rats, along with Wistar rats, are the most common animals used in research and microsurgical training in plastic surgery. These rats have contributed to the development of research in surgical theory and the improvement of surgical techniques (9). In the lymphatic system of the SD rat, Suami et al. clearly depicted the forms and distribution of skin lymphatic vessels of the forelimbs, as well as the connected sentinel lymph nodes (namely, the axillary and brachial lymph lymphosomes), using dye injection (10). However, more accurate anatomical knowledge is required to understand lymphatic circulation and networks. In practice, the vascularized lymphosomes in the rats have been utilized in various studies, for the purpose of; (1) developing animal models of lymphedema, such as simulating impaired lymphatic fluid return (11, 12) (2) investigating the pathophysiological characteristics of vascularized lymph nodes, such as the time to ischemic tolerance and release of associated factors after transplantation (13, 14); and (3) exploring the mechanisms and pathways of vascularized LNFs, such as observing spontaneous lymphovascular anastomosis and lymph node pumping (15). Regardless of the application, a deeper understanding of lymphosome characteristics and dissecting techniques is critical. Since minimal information is available regarding lymphosomes in the rat's forelimb, this study was modified to reveal the physical characteristics and anatomical relationships between ALNFs and BLNFs.

The present study emphasized the reduction of surgical damage and operating time for the dissection process. the most critical anatomical landmark of the ALNFs is the lateral border of the pectoralis major muscle, whereas the exploration space for the BLNFs is located between the triceps and latissimus dorsi muscles. In addition, the operations must be performed under direct microscopic vision, especially for the surgical approach we utilized in ALNFs dissection. Since the vascular pedicles of ALNFs are adjacent to the source artery and surrounded by the branches of the brachial plexus nerve, dissection on the superficial surface of the pectoralis major muscle is preferred. On the other hand, after identifying the latissimus dorsi muscle, a portion of the muscle should be cut off to release pedicles during BLNFs dissection, since the vascular pedicles of BLNFs are longer and found in a deeper location. Because of the length and location of the pedicle in BLNFs, the longer dissection time required for ALNFs indicates that ALNFs dissection involves a more complex procedure. The dissection of the left side of the BLNFs and ALNFs is more difficult than that of the right one, and the proximity of the left LNFs to a few millimeters from the cardiac cavity may affect micromanipulation, due to heart pulsation and the deeper location of the LNFs itself (16).

The physical characteristics of LNFs and their operation time are important for an animal model, in the context of vascularized lymph node transfer. Except for the length of the vascular pedicle, the area and the number of lymph nodes on ALNFs should exceed those on BLNFs when producing the vascularized lymph node flaps in the rodent model. As the pedicle diameters of the two LNFs were not significantly different, both the lateral and dorsal thoracic arteries may be related to the axillary artery, as they are in proximity to one another. Moreover, several studies have shown that the number of lymph nodes is directly proportional to the drainage function of the flap (17, 18). Hence, the right ALNF is the recommended first choice as the donor lymph node flap, followed by the left ALNF. Likewise, if the vessel to be anastomosed is distant from the recipient area, the right BLNF should be selected before the left BLNF is considered. The two LNFs can be combined as a flow–through LNF for complex lymph node reconstruction, where this procedure was deemed feasible–despite not yet having been tested in any study.

A few limitations are present in this study. First, cadaveric studies were not performed. Only data from fresh specimens were obtained; however, fresh specimens can be inadequate for morphological studies. Second, an objective error in the measurement was present. Although we performed the measurement using the imaging software, specimens were subject to tissue shrinkage once harvested (19). Finally, quantitative evaluation of the lymphatic vessels, as well as the functional study of the flap itself, were missing and studies were conducted only on specific rodent animal species (SD rats). Therefore, in future studies, these limitations should be addressed. Future studies should also aim to examine other lymphosomes in rats (18, 19).

To summarize, this study demonstrates the approach to LNF dissection and the anatomical features of the LNFs in the forelimb of the SD rats. In addition to detailed physic characteristics (which included the LNF area, number of lymph nodes, length, and diameter of vascular pedicles), key steps of dissection and the surgical time required for dissection were also described. The anatomical information may be used in various research to modify procedures for vascularized lymph node transfer or lymphovenous anastomosis. Regardless of this study's limitations, these results have the potential to be helpful in producing forelimb LNFs in rodent animal models.

## Data availability statement

The original contributions presented in the study are included in the article/Supplementary material, further inquiries can be directed to the corresponding authors.

## Ethics statement

This study was reviewed and apporoved by the Institutional Animal Care and Use Committee (IACUC) Asan Institute for Life Sciences, Asan Medical Center. The Committee abides by the Institute of Laboratory Animal Resources (ILAR) guide.

## Author contributions

LC: study design, animal operation, data acquisition, analysis, interpretation, and manuscript drafting. JY: data analysis and interpretation and manuscript review. SK: experimental scheduling and animal purchasing and technical and material support. MG: technical and material support. PW: study guidance. JJ: revision for statistical analysis, revised data analysis and interpretation, overall revision, and improvement the manuscript. HC: study design and guidance, data analysis and interpretation, experimental scheduling, funding acquisition, revised all figures, and overall revision of the manuscript. The final manuscript has been read and approved by all authors.

## Funding

This work was supported by the National Research Foundation of Korea (NRF) grant funded by the Korean government (MSIT) (Nos. NRF-2021R1F1A1056527 and NRF-2019R1A2C1009055).

## Conflict of interest

The authors declare that the research was conducted in the absence of any commercial or financial relationships that could be construed as a potential conflict of interest.

## Publisher's note

All claims expressed in this article are solely those of the authors and do not necessarily represent those of their affiliated organizations, or those of the publisher, the editors and the reviewers. Any product that may be evaluated in this article, or claim that may be made by its manufacturer, is not guaranteed or endorsed by the publisher.
